# Probing the early upper respiratory responses to SARS‐CoV‐2

**DOI:** 10.14814/phy2.14836

**Published:** 2021-05-15

**Authors:** Amanda M. Jamieson

**Affiliations:** ^1^ Division of Biology and Medicine Department of Molecular Microbiology and Immunology Brown University Providence RI USA

Since the emergence of the novel respiratory coronavirus, designated SARS‐CoV‐2 in 2019, there have been more than 2.5 million deaths worldwide, and the numbers continue to rise (Zhu et al., 2020). Symptoms from infection by SARS‐CoV‐2, range from entirely asymptomatic to severe acute respiratory distress syndrome (ARDS) (Wiersinga et al., 2020) (Yuki et al., 2020). Severe cases of COVID‐19, the disease caused by SARS‐CoV‐2, are complicated by ARDS and hyper‐inflammation, with many patients requiring mechanical ventilation and ICU admission due to hypoxia (Wiersinga et al., 2020) (Edler et al., 2020). Much of what we know about the disease comes from data from hospitalized patients, who have most likely been infected for a long time and often have severe symptoms. Understanding how SARS‐CoV‐2 infection influences the immune response early in the course of infection is of upmost importance to fully understand how the infection progresses.

The manuscript by Liou et al. (2020) uses an innovative approach to examine the impact of early SARS‐CoV‐2 infection on the innate immune response in the upper respiratory system. By using nasal pharyngeal swab biospecimens from both SARS‐CoV‐2 positive patients and SARS‐CoV‐2 negative patients, the authors are able to determine the impact of a probable early infection on several factors that are important in the early response. In addition, by using both transcriptional and proteomic analysis, this study demonstrates how SARS‐CoV‐2 infection may influence both transcriptional and post‐transcriptional regulation of inflammatory mediators.

One of the most striking findings of the study is that there is a clear correlation of IP‐10 (CXCL10) expression with viral load not only at the transcript level but also at the protein level. CXCL10 expression in the upper respiratory tract has been shown in previous studies to be a hallmark of a respiratory infection (Landry & Foxman, 2018). This chemokine attracts several different immune cell types to the site of infection including macrophages, Natural Killer cells, and activated T cells (Liu et al., 2011). CXCL10 is well known as a factor in ARDS progression, and it has been shown to be elevated in the lungs of patients infected with highly pathogenic avian influenza (HPAI) and SARS‐CoV‐1 (Jiang et al., 2005) (Peiris et al., 2004). Interestingly, it not only has chemotactic activity, but also controls apoptosis, cell growth and proliferation, and angiogenesis (Liu et al., 2011). CXCL10 has been shown to contribute to oxidative burst as well as chemotaxis of activated neutrophils in models of lung injury (Ichikawa et al., 2013). In COVID‐19 patients a biomarker of disease severity is elevated levels of CXCL10 in the plasma (Yang et al., 2020) (Chen et al., 2020). Several previous studies using different models have also shown that nasal epithelial cells express CXCL10 (Gamage et al., 2020) (Lieberman et al., 2020).

Another finding is that in many cases the protein levels do not correspond to what is seen at the mRNA level for the various inflammatory mediators looked at. However, proteins for CXCL10 and several interferon stimulated genes (ISGs) did correlate with transcript level. Further studies are necessary to determine mechanisms behind this interesting observation. It is unclear is this is due to a lack of translation or an active degradation process. Also, it is unknown if this a specific feature of SARS‐CoV‐2 infection where the virus is actively involved in the regulation of host inflammatory mediators, or if this is part of the host process in the tight regulation of the innate immune response during early infection.

This study gives important insight into the early immune and anti‐viral responses to initial SARS‐CoV‐2 infection of the upper respiratory tract. Understanding these early stages of infection will allow us to more fully understand the pathology of COVID‐19.

## References

[phy214836-bib-0001] Chen, Y. , Wang, J. , Liu, C. , Su, L. , Zhang, D. , Fan, J. , Yang, Y. , Xiao, M. , Xie, J. , Xu, Y. , Li, Y. , & Zhang, S. (2020). IP‐10 and MCP‐1 as biomarkers associated with disease severity of COVID‐19. Molecular Medicine, 26, 97. 10.1186/s10020-020-00230-x 33121429 PMC7594996

[phy214836-bib-0002] Edler, C. , Schröder, A. S. , Aepfelbacher, M. , Fitzek, A. , Heinemann, A. , Heinrich, F. , Klein, A. , Langenwalder, F. , Lütgehetmann, M. , Meißner, K. , Püschel, K. , Schädler, J. , Steurer, S. , Mushumba, H. , & Sperhake, J.‐P. (2020). Dying with SARS‐CoV‐2 infection—an autopsy study of the first consecutive 80 cases in Hamburg, Germany. International Journal of Legal Medicine, 134, 1275–1284. 10.1007/s00414-020-02317-w 32500199 PMC7271136

[phy214836-bib-0003] Gamage, A. M. , Tan, K. S. , Chan, W. O. Y. , Liu, J. , Tan, C. W. , Ong, Y. K. , Thong, M. , Andiappan, A. K. , Anderson, D. E. , Wang, D. Y. , & Wang, L.‐F. (2020). Infection of human Nasal Epithelial Cells with SARS‐CoV‐2 and a 382‐nt deletion isolate lacking ORF8 reveals similar viral kinetics and host transcriptional profiles. PLoS Path, 16, e1009130. 10.1371/journal.ppat.1009130 PMC7746279 33284849

[phy214836-bib-0004] Ichikawa, A. , Kuba, K. , Morita, M. , Chida, S. , Tezuka, H. , Hara, H. , Sasaki, T. , Ohteki, T. , Ranieri, V. M. , dos Santos, C. C. , Kawaoka, Y. , Akira, S. , Luster, A. D. , Lu, B. , Penninger, J. M. , Uhlig, S. , Slutsky, A. S. , & Imai, Y. (2013). CXCL10‐CXCR3 enhances the development of neutrophil‐mediated fulminant lung injury of viral and nonviral origin. American Journal of Respiratory and Critical Care Medicine, 187, 65–77. 10.1164/rccm.201203-0508OC 23144331 PMC3927876

[phy214836-bib-0005] Jiang, Y. , Xu, J. , Zhou, C. , Wu, Z. , Zhong, S. , Liu, J. , Luo, W. , Chen, T. , Qin, Q. , & Deng, P. (2005). Characterization of Cytokine/Chemokine profiles of severe acute respiratory syndrome. American Journal of Respiratory and Critical Care Medicine, 171, 850–857. 10.1164/rccm.200407-857OC 15657466

[phy214836-bib-0006] Landry, M. L. , & Foxman, E. F. (2018). Antiviral response in the nasopharynx identifies patients with respiratory virus infection. The Journal of Infectious Diseases, 217, 897–905. 10.1093/infdis/jix648 29281100 PMC5853594

[phy214836-bib-0007] Lieberman, N. A. P. , Peddu, V. , Xie, H. , Shrestha, L. , Huang, M.‐L. , Mears, M. C. , Cajimat, M. N. , Bente, D. A. , Shi, P.‐Y. , Bovier, F. , Roychoudhury, P. , Jerome, K. R. , Moscona, A. , Porotto, M. , & Greninger, A. L. (2020). In vivo antiviral host transcriptional response to SARS‐CoV‐2 by viral load, sex, and age. PLoS Biology, 18, e3000849. 10.1371/journal.pbio.3000849 32898168 PMC7478592

[phy214836-bib-0014] Liou, T. G. , Adler, F. R. , Cahill, B. C. , Cox, D. R. , Cox, J. E. , Grant, G. J. , Hanson, K. E. , Hartsell, S. C. , Hatton, N. D. , Helms, M. N. , Jensen, J. L. , Kartsonaki, C. , Li, Y. , Leung, D. T. , Marvin, J. E. , Middleton, E. A. , Osburn‐Staker, S. M. , Packer, K. A. , Shakir, S. M. , … Sturrock, A. B. (2020). SARS‐CoV‐2 innate effector associations and viral load in early nasopharyngeal infection. medRxiv, 10.1101/2020.10.30.20223545 PMC7903990 33625796

[phy214836-bib-0008] Liu, M. , Guo, S. , Hibbert, J. M. , Jain, V. , Singh, N. , Wilson, N. O. , & Stiles, J. K. (2011). CXCL10/IP‐10 in infectious diseases pathogenesis and potential therapeutic implications. Cytokine & Growth Factor Reviews, 22(3):121–130. 10.1016/j.cytogfr.2011.06.001 21802343 PMC3203691

[phy214836-bib-0009] Peiris, J. , Yu, W. , Leung, C. , Cheung, C. , Ng, W. , Nicholls, J. , Ng, T. , Chan, K. , Lai, S. , Lim, W. , Yuen, K. , & Guan, Y. (2004). Re‐emergence of fatal human influenza A subtype H5N1 disease. The Lancet, 363, 617–619. 10.1016/S0140-6736(04)15595-5 PMC7112424 14987888

[phy214836-bib-0010] Wiersinga, W. J. , Rhodes, A. , Cheng, A. C. , Peacock, S. J. , & Prescott, H. C. (2020). Pathophysiology, transmission, diagnosis, and treatment of coronavirus disease 2019 (COVID‐19): A review. JAMA, 324, 782. 10.1001/jama.2020.12839 32648899

[phy214836-bib-0011] Yang, Y. , Shen, C. , Li, J. , Yuan, J. , Wei, J. , Huang, F. , Wang, F. , Li, G. , Li, Y. , Xing, L. , Peng, L. , Yang, M. , Cao, M. , Zheng, H. , Wu, W. , Zou, R. , Li, D. , Xu, Z. , Wang, H. , … Liu, Y. (2020). Plasma IP‐10 and MCP‐3 levels are highly associated with disease severity and predict the progression of COVID‐19. Journal of Allergy and Clinical Immunology, 146, 119–127.e4. 10.1016/j.jaci.2020.04.027 PMC7189843 32360286

[phy214836-bib-0012] Yuki, K. , Fujiogi, M. , & Koutsogiannaki, S. (2020). COVID‐19 pathophysiology: A review. Clinical Immunology, 215, 108427. 10.1016/j.clim.2020.108427 32325252 PMC7169933

[phy214836-bib-0013] Zhu, N. , Zhang, D. , Wang, W. , Li, X. , Yang, B. , Song, J. , Zhao, X. , Huang, B. , Shi, W. , Lu, R. , Niu, P. , Zhan, F. , Ma, X. , Wang, D. , Xu, W. , Wu, G. , Gao, G. F. , & Tan, W. (2020). A Novel coronavirus from patients with pneumonia in China, 2019. New England Journal of Medicine, 382, 727–733. 10.1056/NEJMoa2001017 PMC7092803 31978945

